# National Cancer Institute Centers With Environmental Sustainability Plans for Climate Change

**DOI:** 10.1001/jamanetworkopen.2023.17206

**Published:** 2023-06-20

**Authors:** Katie E. Lichter, Hannah N. W. Weinstein, Marium Husain, Raina Kishan, Andrew Hantel, Ashray Maniar

**Affiliations:** 1 University of California, San Francisco; 2 Columbia University, New York, New York; 3 The Ohio State University Wexner Medical Center, Columbus; 4 Mount Sinai Hospital, New York, New York; 5 Dana-Farber Cancer Institute, Boston, Massachusetts; 6 Kaiser Permanente Northwest, Portland, Oregon

## Abstract

This qualitative study investigates environmental sustainability plans at National Cancer Institute Comprehensive Cancer Centers and affiliated institutions.

## Introduction

Climate change poses significant risks for cancer incidence, care delivery, and outcomes. Environmental exposures related to climate change, such as air pollution, particulate matter, and ultraviolet radiation, are associated with increased cancer incidence and cancer-specific mortality.^1,2^ The US health care industry is responsible for approximately 8.5% of the country’s greenhouse gas (GHG) emissions, which are partly attributable to cancer centers and associated with a loss of 388 000 disability-adjusted life-years annually.^3^ Cancer centers and their patients are at particularly high risk from the impacts of climate change, with care at risk of disruption and high-risk communities experiencing disproportionate outcomes.^4,5^

As the climate shifts, it is imperative that cancer centers address and prepare for delivering high-quality, equitable care. Establishing emergency preparedness plans to provide care during climate-fueled disasters and developing environmental sustainability plans to reduce the environmental footprint of cancer centers are urgently needed for adaptation and mitigation. Sustainability plans typically address US Environmental Protection Agency (EPA) GHG Protocol scopes:^6^ (1) direct GHG emissions from health care (eg, facilities, anesthetics, and vehicles), (2) indirect GHG emissions from purchased energy (eg, electricity), and (3) other indirect emissions (eg, staff commuting and the supply chain for medicines and medical devices). Of note, this category accounts for more than 80% of health care emissions.^6^

This study reports on publicly available environmental sustainability plans at National Cancer Institute (NCI)–designated Comprehensive Cancer Centers (hereafter, *Centers*) and affiliated universities and hospitals.

## Methods

Columbia University determined that informed consent and study approval were not needed for this qualitative study given its use of deidentified data and institutional review board exemption per 45 CFR §46.104. The study follows the Strengthening the Reporting of Observational Studies in Epidemiology (STROBE) reporting guideline.

Centers and affiliated hospitals and universities were investigated September to December 2021. Environmental sustainability plans were identified through a structured search engine query (Google): (“[Center name] AND [climate action plan OR climate change OR sustainability plan OR carbon neutral OR green]”), with review of the first 20 results. Websites of the Center and affiliated hospital and university were then manually reviewed, repeating the previously described search in an embedded search function if available. Plans or programs identified were abstracted and assessed via the existence of US EPA GHG Protocol scope 1 to 3 emissions. Key sustainability personnel and hospital leadership identified online or through Center administration were contacted via email to obtain additional information. Search and review processes were completed by sets of 2 investigators (K.E.L., M.H., R.K., and A.M.) independently.

## Results

Among 64 NCI-designated Centers, 2 Centers (3.1%) had independent sustainability plans that included EPA scopes 1 to 3 and 11 Centers (17.2%) reported on their environmental sustainability efforts (Table; Figure). A total of 54 affiliated hospitals and universities (84.4%) had environmental sustainability plans (10 hospitals, 28 universities, and 16 medical centers) (Table). Of affiliated hospitals and universities with environmental sustainability plans, 5 institutions (7.8%) included the Center in plans (Table). Most affiliated hospitals and universities with plans (49 institutions [76.6%]) had leadership or personnel (eg, a medical director of sustainability or hospital green team) responsible for sustainability efforts.

**Table.** Environmental Sustainability Plans at Cancer Centers

Queried information	NCI-designated Comprehensive Cancer Centers (N = 64) No. (%)
Centers with stand-alone environmental sustainability plans	2 (3.1)
Centers reporting on climate and environmental sustainability programs	11 (17.2)
Affiliated hospitals, universities, and medical centers with environmental sustainability plans	54 (84.4)^a^
Affiliated hospitals and universities with environmental sustainability plans that include the cancer center	5 (7.8)
Affiliated hospitals and universities with appointed environmental sustainability leadership or personnel	49 (76.6)

Abbreviation: NCI, National Cancer Institute.

^a^ Of 54 affiliated hospitals and universities with environmental sustainability plans, there were 10 hospitals, 28 universities, and 16 medical centers (combined hospitals and universities).

**Figure.**
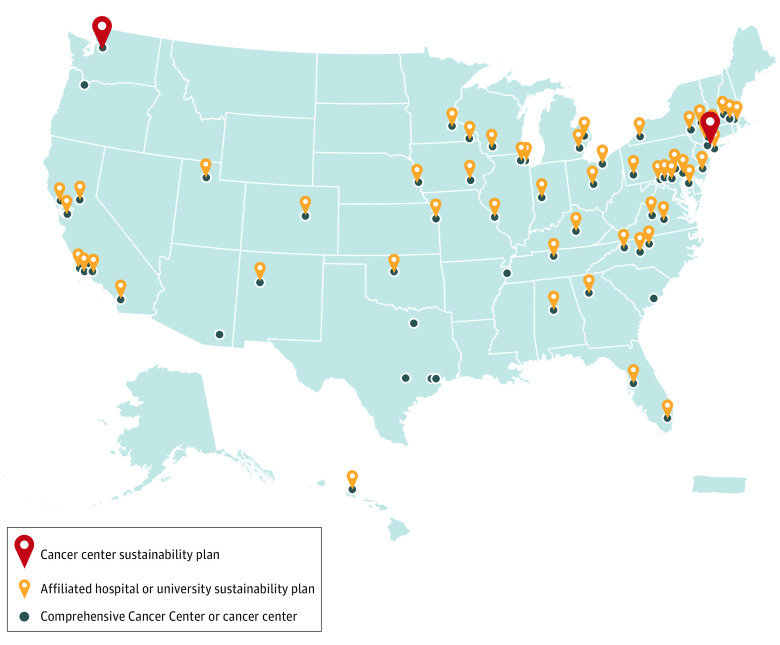
Map of Environmental Sustainability Plans at National Cancer Institute–Designated Centers

## Discussion

Limitations of this qualitative study are mainly associated with the restricted information accessible through online sources and researchers’ inability to authenticate information from personnel. As the impacts of climate change on public health become increasingly evident, it is essential that oncology Centers prioritize the implementation of adaptation and mitigation goals as outlined in the Health Sector Climate Pledge. This study found that there was a critical gap in the readiness of Centers to address climate change and engage in environmental sustainability planning and reporting. However, most affiliated hospitals and universities (84.4%) had accessible sustainability plans and actions in place, which may provide a significant opportunity for NCI-designated Centers to partner with affiliated organizations and take collective action to mitigate climate-associated outcomes for patients and the planet.

## Notes

Supplement.
Data Sharing Statement
