# Supplementary material for: National Cancer Institute Centers With Environmental Sustainability Plans for Climate Change
Source: JAMA Netw Open. 2023 Jun 20;6(6):e2317206. doi: 10.1001/jamanetworkopen.2023.17206 (PMC10282889; doi:10.1001/jamanetworkopen.2023.17206)
Supplement: Supplement. — Data Sharing Statement [file jamanetwopen-e2317206-s001.pdf]

Data Sharing Statement

Lichter. National Cancer Institute Centers With Environmental Sustainability Plans for Climate Change. *JAMA Netw Open*. Published June 20, 2023.
doi:10.1001/jamanetworkopen.2023.17206

Data
Data available: Yes
Data types: Data (not involving human participants)
How to access data: Data available via request to author Katie Lichter (katie.lichter@ucsf.edu)
When available: With publication

Supporting Documents
Document types: None

Additional Information
Who can access the data: Anyone requesting the data.
Types of analyses: For any purpose.
Mechanisms of data availability: N/a
Any additional restrictions: N/a
